# Validity and reliability of evaluating hip abductor strength using different normalization methods in a functional electromechanical device

**DOI:** 10.1371/journal.pone.0202248

**Published:** 2018-08-20

**Authors:** Enrique Cerda Vega, Daniel Jerez-Mayorga, Ramón Machado Payer, Christian Campos Jara, Iris Guzman-Guzman, Alvaro Reyes Ponce, Luis Javier Chirosa

**Affiliations:** 1 Carrera de Kinesiología, Departamento Ciencias de la Salud, Facultad de Medicina, Pontificia Universidad Católica de Chile, Santiago, Chile; 2 Facultad Ciencias de la Rehabilitación, Universidad Andrés Bello, Santiago, Chile; 3 Faculty of Sciences for Physical Activity and Sport, University of Granada, Granada, Spain; 4 Unidad Académica de Ciencias Químico-Biológicas, Universidad Autónoma de Guerrero, Chilpancingo, México; Universite de Nantes, FRANCE

## Abstract

The hip abductor muscles are vitally important for pelvis stability, and common strength deficits can negatively affect functionality. The muscle strength can be measured using different dynamometers and be evaluated in three positions (side-lying, standing, and supine). Obtained strength data can be expressed in different ways, with data normalization providing more objective and comparable results. The aim of this study was to establish the validity and reliability of three protocols in evaluating the isometric strength of the hip abductor muscles. A new functional electromechanical dynamometer assessed strength in three positions, with findings subjected to three data normalization methods. In two identical sessions, the hip abductor strengths of 29 subjects were recorded in the side-lying, standing, and supine positions. Peak force was recorded in absolute terms and normalized against body mass, fat-free mass, and an allometric technique. The peak force recorded in the side-lying position was 30% and 27% higher than in the standing and supine positions, respectively, independent of data normalization methodology. High inter-protocol correlations were found (*r*: 0.72 to 0.98, *p* ≤ 0.001). The supine position with allometric data normalization had the highest test-retest reliability (0.94 intraclass correlation coefficient and 5.64% coefficient of variation). In contrast, the side-lying position with body mass data normalization had a 0.66 intraclass correlation coefficient and 9.8% coefficient of variation. In conclusion, the functional electromechanical dynamometer is a valid device for measuring isometric strength in the hip abductor muscles. The three assessed positions are reliable, although the supine position with allometric data normalization provided the best results.

## Introduction

The hip abductor muscles are key in stabilizing the pelvis. This is particularly so for unipedal stances, such as walking, [1]. In conjunction with these muscles, the biomechanical properties of the joints must be prepared to receive heavy loads and ensure mobility of the inferior limbs and trunk. All of these factors highlight the importance of this zone in maintaining stability during daily tasks and sporting activities that involve unipedal impacts [2, 3].

Strength deficits in the hip abductor muscles occur as a result of aging and certain pathologies, thus negatively affecting daily life activities [4]. Pathologies such as hip injuries, including osteoarthritis [5, 6] and complete/partial joint replacement [7], not only affect the strength of the injured limb, but also of the contralateral limb [8]. Consequent impacts to walking can include the Trendelenburg gait [9], although dysfunctions can arise distant to the affected joint, including lower back dysfunctions [10] and patellofemoral pain syndrome in the knee [11–16].

Maintenance of optimum isometric strength in the hip muscles has been linked to clinical and functional improvements in athletes and patients with musculoskeletal conditions [17–19]. Therefore, understanding the role of hip muscles in abduction movements would facilitate the diagnosis and effective treatment of alterations caused within the inferior extremities [20]. In clinical settings, hip abduction strength is primarily assessed through three procedures, i.e. manual muscle testing, isokinetic dynamometry, and hand-held dynamometry. The isokinetic dynamometer is an exact, secure evaluation tool and the current gold standard for assessing muscle strength. Nevertheless, the high cost of this instrument limits accessibility [21, 22]. In turn, manual muscle testing has severe reliability limitations and is reliant on the experience of the evaluator [23]. Finally, while the manual dynamometer is low-cost, accessible, and validated for muscle assessments, this method is dependent on external adjustments to improve result validity and reliability [24–26].

These three methods have been assessed using three positions, i.e. the side-lying position (SlP), supine position (SupP), and standing position (StP); however, the SlP is the only position validated for all three methods [27–36]. Despite this, the StP has been described as the best physiologically and functionally as most functional tasks are performed in this position [27, 37]. Similarly, the SupP has been favorably cited as maintaining neutral gravity and for preventing problems caused by supporting the body on one side [30]. These different positions provide alternatives for subjects that cannot be in one position or another due to health issues.

Also worth considering in relation to strength measurements are the various ways in which results can be expressed. These variations are due to variables that influence strength, such as body mass and muscle mass. Therefore, data must be normalized to prevent the effects of these variables on the final results [38, 39]. Similarly, a measurement independent of body mass is needed so that individuals can be compared against others and with themselves between measurements.

To this end, a new dynamometric device was recently designed for the assessment of functional tasks. This instrument allows evaluating movements in different planes and at different angles through a pulley system, which permits specific, natural movements [40, 41]. This so-termed functional electromechanical dynamometer can be used for static and dynamic assessments, including isokinetic evaluations in different muscle groups [41].

The aim of this study was to determine the validity and reliability of evaluation protocols for isometric strength in the hip abductor muscles of healthy subjects. Specifically, the functional electromechanical dynamometer was used to evaluate the hip abductor muscles in a SlP, SuP, and StP, and data were then assessed with three normalization methods.

## Material and methods

This was a descriptive study with non-probability sampling using a group of volunteers. To investigate test-retest validity and reliability, the isometric strength of the hip abductor muscles was analyzed in two identical sessions separated by at least 48 h. Both sessions were completed by all participants within a ten day period. The same researcher took all measurements, ensured identical conditions for all assessments/sessions, and provided volunteers with identical instructions.

### Participants

In January 2017, physical therapy students of the Pontificia Universidad Católica were invited to participate. Twenty-nine volunteers (14 males, 15 females) accepted to participate and none of them dropped out. The volunteers presented the following average traits: 20.7 ± 1.8 years-old; 66.7 ± 13.9 kg weight; 169.4 ± 8.4 cm height; 23.1 ± 3.37 body mass index; 51.9 ± 10.7 kg Fat Free Mass; 15.1 ± 6.9 kg fat mass and 21,7% ± 7,4 percentage of body mass. All healthy volunteers were aged between 18 and 25, presented no cardiovascular, lung, or metabolic pathologies. They all reported no musculoskeletal pain within the three months prior to assessments and they practiced physical activity at least twice a week as part of their academic training. All procedures were approved by the Ethical Committee of the Faculty of Medicine, Pontificia Universidad Católica de Chile (CEC-MedUC 16–399) and were in accordance with the 2013 Helsinki Declaration. Individuals in this manuscript have given written informed consent (as outlined in PLOS consent form) to publish these case details.

### Procedure

Before any evaluations were performed, all procedures were verbally explained to the volunteers, who were then required to provide signed informed consent before participating. All participants also filled out a personal information sheet and responded to the Physical Activity Readiness Questionnaire [42]. Anthropomorphic measurements were then taken, including weight (kg), height (cm), and body composition through bioelectrical impedance analysis (Bodystat, Quadscan 4000) following procedures described by Lukaski [43], from which of fat-free mass (FFM) and fat mass were obtained (Kg).

Participants warmed up for 10 min on an ergometer bicycle (FitPro CU 800) at an intensity of 50% maximum heart rate, after that they followed 3 submaximal repetitions of 20 seconds for each of the positions (ie, SlP, StP and SupP). For subsequent abduction assessments, volunteers were given the following instructions according to Bemben M. et al [44]: perform abduction of the extremity, exerting the maximum contraction possible as quick as possible. These instructions allow to obtain the highest PF values [44]. Then, the volunteers were asked to exert and hold maximum isometric contractions for 6 s, with three alternating repetitions performed in non-dominant side with 1 minute of rest after each repetition. This procedure was repeated for each of the three assessed positions. Participants were allowed to rest for 10 min between each assessed position, and the order in which positions were evaluated was random. The strength exerted for each maximum isometric contraction was measured using the Haefni Health v.1.0 electromechanical dynamometer (iVolution R&D, Granada, Spain), which has been validated for this use [40, 41]. During task execution, subjects were motivated to exert maximum force by the evaluator saying: “let’s go, let’s go, come on, come on.”

For assessments in the SlP, participants laid down on a stretcher, resting against their contralateral side. Patients were then asked to bend their contralateral knee 90° to improve stability, and a foam wedge was placed between both legs to maintain alignment of the extremity under evaluation at 0° of abduction (i.e. neutral position). A fixing strap was then placed at the level of the iliac crests, thereby firmly holding the subject’s pelvis against the stretcher. Resistance was placed at the extreme distal end of the extremity under evaluation, 1 cm above the lateral malleolus. For assessments in the StP, participants stood in front of the stretcher. A foam wedge was placed on the stretcher and was used by subjects to rest their hands, which were at the level of the iliac crests. Participants were instructed not to exert any force with their hands. The volunteer’s feet were separated at a distance equal to their shoulders. Resistance was placed 1 cm above the lateral malleolus. Finally, for assessments in the SupP, participants laid down on their back. A fixing strap was placed at the level of the iliac crests, thereby firmly holding the subject to the stretcher. The lower extremities were at 0° of abduction, and the upper extremities were crossed against the thorax. Resistance was placed at the extreme distal end of the extremity under evaluation, 1 cm above the lateral malleolus.

Test results were automatically stored in the Haefni Health device and were not revealed to the subjects or evaluator at the time of task execution. Once all measurements were taken, data were extracted to Excel format using the Haefni Health device software. For posterior analysis peak force (PF) values were expressed in absolute terms in Newtons (N), by following the allometric technique hip muscle method described by Brazet-Jones et al. [38] by applying this method we devided the PF by an exponential body mass (BM) specifically differentiated for men and for women (0.792 and 0.482 respectively), by the ratio between PF and BM (PF/BM) and by the ratio between PF and FFM (PF/FFM).

### Statistical analysis

Data was initially evaluated for normality using the Shapiro-Wilk test. The *t* test for independent samples was performed to determine test-retest differences. Descriptive statistics (mean and standard deviation) were used to describe PF and anthropomorphic data. To determine the degree of linear association between the different positions, the Pearson coefficient of correlation was used, with significance established at *p* ≤ 0.05. The coefficient of correlation was interpreted through classifications described by Mukaka [45], where 0,9 to 1,0 was very high correlation, 0,7 to 0,9 was high, 0,5 to 0,7 was moderate, 0,3 to 0,5 was low, and 0,00 to 0,3 was negligible correlation. To determine test-retest reliability of the hip abductor muscles, a one-way analysis of variance was used to calculate the intraclass coefficient of correlation (ICC) with a 95% confidence interval [46]. The classification system established by Koo et al. [47] was used, where an ICC < 0.5 was poor, 0.5–0.75 was moderate, 0.75–0.9 was good, and > 0.9 was excellent. Absolute reliability was determined using the coefficient of variation (CV) [48], where < 10% was considered good [49]. The standard error of the mean (SEM) was established following Eliasziw et al. [50]. Differences between test-retest and average values were graphically assessed using a Bland-Altman plot [51] with a 95% confidence interval and the smallest detectable difference (SDD) was calculated with a 95% confidence as described by Weir JP.[52]. All statistical tests were executed in the Stata v.9.0 software, while the Graphpad software was used for figure construction.

## Results

The data showing that the normality assumption was met for all variables included in the study. There were no differences between test and retest in all positions and in all the other measurements. The highest maximum isometric strength values, obtained via functional electromechanical device in the test and retest, were in the SlP, independent of the method used for data presentation (Table 1). Furthermore, significant high test-retest correlations (*r* = 0.78 to 0.92, *p* < 0.001) were found for all positions (Table 2).

**Table 1:** Peak force values (test and restest) in different assessment positions and as normalized by different methods.

Position	Measure	PF	PF/BJ	PF/BM	PF/FFM
***SlP***	Test	228.1±57.2	37.7±15.8	3.5±0.8	2.7±0.7
	Retest	224.4±58.3	36.5±14.1	3.4±0.6	2.7±0.7
***StP***	Test	164.9±46.9	26.7±9.9	2.5±0.5	2.0±0.6
	Retest	158.3±38.6	25.7±9.3	2.4±0.4	1.9±0.5
***SupP***	Test	162.8±41.9	26.9±11.1	2.5±0.5	2.0±0.5
	Retest	164.2±44.8	26.7±9.8	2.5±0.5	2.0±0.5

Abbreviations: PF, peak force (N); PF/BJ: normalized by Brazet-Jones et al. [38]; PF/BM: normalized by body mass; PF/FFM: normalized by fat-free mass; SlP, side-lying position; StP, standing position; SupP: supine position. Data are shown as the mean ± standard deviation.

**Table 2:** Test-retest correlations for peak force in the different assessed positions.

*Strength Measurement*	*SlP* *Test vs retest* r*(p)	*StP* *test vs retest* r* (p)	*SupP* *test vs retest* r*(p)
**PF**	0.78 (<0.001)	0.91 (<0.001)	0.92 (<0.001)

Abbreviations: PF, peak force (N); SlP, side-lying position; StP, standing position; SupP, supine position. r* Pearson correlation coefficient. Statistical significance was established at p < 0.05.

A high linear relationship existed between positions (e.g. SlP vs StP; SlP vs SupP; StP vs SupP) independent of how data were expressed (Table 3). The highest correlations were obtained when data were normalized using the Brazet-Jones method (*r* = 0.93 to 0.98, *p* < 0.001). Nevertheless, while correlations were high for PF/BM normalization, these values were consistently lower than those obtained via other methods (*r =* 0.74 to 0.90, *p* < 0.001). Furthermore, the highest correlation values were between the StP and SupP for all normalization methods (*r* = 0.90 to 0.98, *p* < 0.001).

**Table 3:** Correlations and significance levels between the different evaluated positions and obtained strength values.

Strength	r* SlP vs *StP*	*p*	r*SlP vs *SupP*	*p*	r* StP vs *SupP*	*p*
PF	0.88	≤ 0.001	0.88	≤ 0.001	0.95	≤ 0.001
PF BJ	0.93	≤ 0.001	0.93	≤ 0.001	0.98	≤ 0.001
PF/BM	0.74	≤ 0.001	0.72	≤ 0.001	0.90	≤ 0.001
PF/FFM	0.88	≤ 0.001	0.88	≤ 0.001	0.95	≤ 0.001

Abbreviations: PF, peak force (N); PF/BJ: normalized by Brazet-Jones et al. [38]; PF/BM: normalized by body mass; PF/FFM: normalized by fat-free mass; SlP, side-lying position; StP, standing position; SupP: supine position. r* Pearson correlation coefficient. Statistical significance was established at p < 0.05.

**Fig 1:**
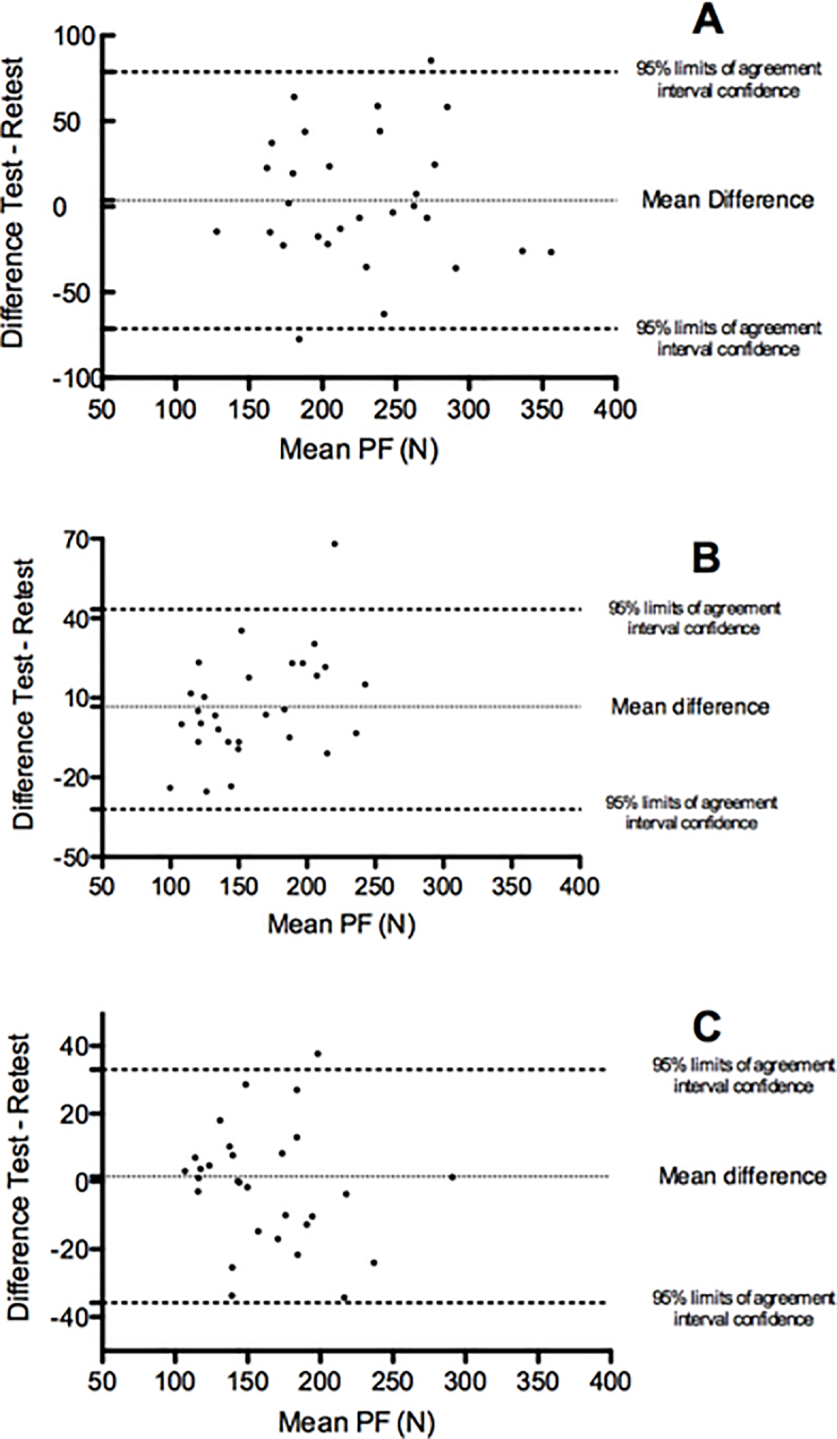
Bland-Altman plot for test-retest and average peak forces in different positions. **(A)** side-lying position (SlP); **(B)** standing position (StP); and **(C)** supine position (SupP).

In turn, reliability was measured using the ICC with a 95% confidence interval (Table 4). The lowest values were found in the SlP (0.66 to 0.78 ICC), whereas the highest values were obtained in the SupP (0.87 to 0.94 ICC). The PF/BJ method was established as the best for data expression (0.88 to 0.94 ICC). The lowest CVs were found in the SupP (5.64%), whereas the highest CVs were recorded in the SlP (9.8%). Similarly, the highest SEM values were found in the SlP, and the lowest SEM values were obtained in the SupP, excepting when data were normalized by the PF/BJ method in the StP. The characteristics of the SEM in the different positions and the different ways of expressing the results were reflected similarly for the SDD. Differences in PF between positions were graphically expressed via a Bland-Altman plot (Fig 1A–1C).

**Table 4:** Reliability measurements for peak force values obtained in the three assessed positions (i.e. SlP, StP, and SupP) and as evaluated by different normalization techniques (i.e. Brazet-Jones, body mass, fat-free mass).

Position	*SlP*						*StP*						*SupP*					
Variable	ICC	95% CI		CV%	SEM	SDD	ICC	95% CI		CV%	SEM	SDD	ICC	95% CI		CV%	SEM	SDD
PF	0.78	0.64	0.92	9.80	23.96	66.41	0.88	0.80	0.96	6.60	14.02	38.86	0.92	0.86	0.97	5.64	11.73	32.51
PF/BJ	0.88	0.80	0.96	9.80	4.89	13.47	0.94	0.91	0.98	6.60	2.29	6.35	0.94	0.91	0.98	5.64	2.50	6.93
PF/BM	0.66	0.46	0.87	9.80	0.33	0.91	0.80	0.68	0.93	6.60	0.18	0.50	0.87	0.78	0.96	5.64	0.16	0.44
PF/FFM	0.77	0.63	0.92	9.80	0.28	0.78	0.88	0.81	0.96	6.60	0.16	0.44	0.92	0.86	0.97	5.64	0.14	0.39

Abbreviations: CI, confidence interval; CV, coefficient of variation; ICC, intraclass correlation coefficient; SDD, smallest detectable difference, PF, peak force (N); PF/BJ: normalized by Brazet-Jones et al. [38]; PF/BM: normalized by body mass; PF/FFM: normalized by fat-free mass; SEM, standard error of measurement, SlP, side-lying position; StP, standing position; SupP: supine position.

## Discussion

The results of this study show that the FED is a valid and reliable instrument to measure the strength of the hip abductor musculature in all evaluated positions. Peak force values were highest when in the SlP, independent of the method used to express results. This finding is relevant as two premises were established for determining the construct validity of hip abduction strength as measured with a functional electromechanical dynamometer. The first premise was that the SlP is a valid position for this assessment [36]. The second premise was that the most valid position for measuring strength would be that in which the highest PF values were obtained, as per the bilateral deficit principle. This principle establishes that the force generated by a muscle will be less than when the contralateral muscle is also used [53]. In assessing the three positions (Table 1), the highest PF values were obtained in the SlP. Indeed, these values were 30% and 26.8% greater than values respectively obtained in the StP and SupP. This finding is in line with Widler et al. [36], a study that applied hand-held dynamometry. As associated with the bilateral deficit principle [53], increasing contralateral muscle requirements, as needed to maintain stability, would decrease the maximum force generated by the muscles under evaluation. Therefore, since the SlP provides greater pelvic stabilization, demands to contralateral muscles would be reduced.

This relationship between PF and position was maintained independent of how data were expressed, whether in absolute values or through any of the three normalization methods used. This contrasts with findings by Widler et al. [36], who found significantly higher PF values in the StP than in the SupP, a result attributed to the lower stabilization provided in the SupP. In the present study, no differences were found in PF between these two positions, due to which, we propose that the fixing strap placed at the level of the iliac crests was sufficient in providing similar stabilization in the StP and SupP. The lack of differences in PF would further indicate that the functional electromechanical dynamometer is equally valid in both positions.

When correlating the PF values obtained in the three positions (Table 3), a high, statistically significant correlation was found between all positions, regardless of data normalization methodology. The best relationship was found between StP and SupP with PF/BJ normalization (*r* = 0.98, *p* ≤ 0.001)[38]. This finding suggests that while these positions result in lower PF values (i.e. less valid), the StP and SupP could be used when testing in the SlP is not possible. In turn, while body mass is one of the most commonly used normalization techniques [39], the presently obtained results indicate that this technique results in the lowest correlations, especially when comparing the SlP and SupP (*r* = 0.72, *p* ≤ 0.001). This might be due to the supposition in PF/BM normalization that greater strength is directly proportional to body mass, which is not always the case [54]. Furthermore, this normalization method does not consider sex or inherent traits of the segment under evaluation. In turn, both of these points are included in the PF/FFM and PF/BJ techniques, which had high to very high correlations (*r* = 0.88 to 0.98, *p* ≤ 0.001). These results support that the SlP is a valid position and that the StP and SupP could be good alternatives in specific cases.

The second objective of this study was related to the reliability of the three utilized protocols. The ICCs were good to excellent for all three positions (Table 4). Nevertheless, the best results were obtained for the SupP, independent of data being expressed in absolute terms or normalized with any of the three applied methods. In assessing the different ways to express the data, the PF/BJ method [38] was consistently the most reliable (0.88 and 0.94 ICCs for SlP and StP/SupP, respectively). These results support the initial findings of Brazet-Jones et al. [38]. Furthermore, Meyer [55] evaluated reliability in the SlP of an isokinetic device equipped with a new stabilization system, the aim of which was to obtain more reliable results. Meyer [55] expressed PF in absolute terms (0.91 ICC) and using the PF/BJ technique (0.96 ICC). These values are similar to the presently obtained ICC values for the SupP (0.92 and 0.94 ICC, respectively), although values in the SlP were comparatively lower (0.78 and 0.88 ICC, respectively). Unfortunately, Meyer [55] did not assess other positions or normalization methods, thus limiting comparisons with the current study. In the case of hand-held dynamometry, Widler et al. [36] used external fixation and compared three positions, also reporting high ICC values (SlP: 0.902, StP: 0.880, and SupP: 0.826). Nevertheless, the data presented by Widler et al. [36] were normalized only as a percentage of body mass, and the presently obtained results showed higher ICC values when normalized by the PF/FFM or PF/BJ methods. Similar results were obtained by Fenter et al. [28], who assessed PF in the SupP with various hand-held dynamometers, reporting ICC values between 0.89 and 0.94. In turn, while Thorborg [56] also used hand-held dynamometry, external fixation was not used. Instead, fixation was exerted by different evaluators in the SupP, resulting in PF values with a 0.84 ICC.

Few studies in the hip muscles have used the CV to determine absolute reliability. In the present study, the CVs were low (SlP: 9.8%, StP: 6.6%, and SupP: 5.64%). These values are in line with that reported by Stokes et al. [49], especially for the SupP. In contrast, Widler et al.[36] reported the lowest CV values for the SlP and StP (3.67% and 4.22%, respectively) and the highest for the SupP (6.11%). Using a similar system, and evaluating only the SlP, Nadler [57] obtained a CV of 4.7%. In relation to SEM values, these were generally low, with the SupP being the best in this regard (11.73 SEM). The SDD allows the clinician to determine the value from which, after a second measurement, it can be considered as a real difference 95% of the time and not a difference attributable to the measurement error. These values are not described in the literature for the 3 positions and forms of normalization with the FED HHe. The SDD depends on the SEM for its calculation, therefore it behaved following a similar pattern. Since the present research is a reliability study, it is expected that the differences among the values obtained between the test and the retest will be lower than the SDD value, which is true for all the positions and ways of delivering the results.

When the data were differentially expressed (i.e. absolute, PF/BM, PF/FFM, or PF/BJ), results normalized using the PF/BJ technique were consistently the most reliable, particularly for the SupP. On the other hand, data were least reliable when expressed using PF/BM normalization. The three protocols used in this study bring possibilities to the specialist to evaluate patients who can not use SlP. This it is specially important to be consider in patients with different severity of hip pathologies and elderly patients. There are restricted protocols to evaluate patients in these conditions so therefore our results may offer an alternative way to evaluate them using a standarized method.

## Conclusions

The Haefni Health functional electromechanical dynamometer is a valid device for measuring isometric strength in the hip abductor muscles. The three assessed protocols were found reliable, although the supine position obtained the best results. Regarding data expression, the technique described by Brazet-Jones et al. [38] was the most reliable. Considering the obtained information, we recommend using the side-lying position when measuring hip abductor strength with a functional electromechanical dynamometer. When this is not possible, the supine position should be preferred. To normalize the resulting data, we recommend applying the methodology described by Brazet-Jones et al. [38], and, in contrast, normalization by body mass should be avoided.

## Supporting information

S1 Dataset Results evaluation with FED. (XLSX)

S1 Fig Syde Lyng position assessment. (JPG)

S2 Fig Standing position assessment. (JPG)

S3 Fig Supine position assessment. (JPG)

S4 Fig Functional electromechanical dynamometer, Haefni Health (HHe). (JPG)

## References

[1] Klemetti R, Steele KM, Moilanen P, Avela J, Timonen J. Contributions of individual muscles to the sagittal- and frontal-plane angular accelerations of the trunk in walking. J Biomech. 2014;47(10):2263–8. 10.1016/j.jbiomech.2014.04.052 . 24873862

[2] Smith JA, Popovich JM Jr., Kulig K. The influence of hip strength on lower-limb, pelvis, and trunk kinematics and coordination patterns during walking and hopping in healthy women. J Orthop Sports Phys Ther. 2014;44(7):525–31. 10.2519/jospt.2014.5028 . 24816500

[3] Lenhart R, Thelen D, Heiderscheit B. Hip Muscle Loads During Running at Various Step Rates. J Orthop Sport Phys. 2014;44(10):766–74. 10.2519/jospt.2014.5575 WOS:000342482900008. 25156044 PMC4296582

[4] Johnson ME, Mille ML, Martinez KM, Crombie G, Rogers MW. Age-related changes in hip abductor and adductor joint torques. Arch Phys Med Rehabil. 2004;85(4):593–7. . 15083435 10.1016/j.apmr.2003.07.022

[5] Dwyer MK, Stafford K, Mattacola CG, Uhl TL, Giordani M. Comparison of gluteus medius muscle activity during functional tasks in individuals with and without osteoarthritis of the hip joint. Clinical biomechanics (Bristol, Avon). 2013;28(7):757–61. Epub 2013/08/06. 10.1016/j.clinbiomech.2013.07.007 . 23911109

[6] Loureiro A, Mills PM, Barrett RS. Muscle weakness in hip osteoarthritis: a systematic review. Arthritis Care Res (Hoboken). 2013;65(3):340–52. 10.1002/acr.21806 . 22833493

[7] Horstmann T, Listringhaus R, Haase GB, Grau S, Mundermann A. Changes in gait patterns and muscle activity following total hip arthroplasty: a six-month follow-up. Clinical biomechanics (Bristol, Avon). 2013;28(7):762–9. 10.1016/j.clinbiomech.2013.07.001 . 23906936

[8] Jaramillo J, Worrell TW, Ingersoll CD. Hip isometric strength following knee surgery. J Orthop Sports Phys Ther. 1994;20(3):160–5. 10.2519/jospt.1994.20.3.160 . 7951293

[9] Hardcastle P, Nade S. The significance of the Trendelenburg test. J Bone Joint Surg Br. 1985;67(5):741–6. . 4055873 10.1302/0301-620X.67B5.4055873

[10] Lazennec JY, Brusson A, Rousseau MA. Hip-spine relations and sagittal balance clinical consequences. Eur Spine J. 2011;20 Suppl 5:686–98. 10.1007/s00586-011-1937-9 ; PubMed Central PMCID: PMCPMC3175930. 21796392 PMC3175930

[11] De Marche Baldon R, Nakagawa TH, Muniz TB, Amorim CF, Maciel CD, Serrão FV. Eccentric Hip Muscle Function in Females With and Without Patellofemoral Pain Syndrome. Journal of Athletic Training. 2009;44(5):490–6. 10.4085/1062-6050-44.5.490 PMC2742458. 19771287 PMC2742458

[12] Barton CJ, Lack S, Malliaras P, Morrissey D. Gluteal muscle activity and patellofemoral pain syndrome: a systematic review. Br J Sports Med. 2013;47(4):207–14. 10.1136/bjsports-2012-090953 . 22945929

[13] Boling MC, Padua DA, Alexander Creighton R. Concentric and Eccentric Torque of the Hip Musculature in Individuals With and Without Patellofemoral Pain. Journal of Athletic Training. 2009;44(1):7–13. PMC2629043. 10.4085/1062-6050-44.1.7 19180213 PMC2629043

[14] Finnoff JT, Hall MM, Kyle K, Krause DA, Lai J, Smith J. Hip strength and knee pain in high school runners: a prospective study. PM R. 2011;3(9):792–801. 10.1016/j.pmrj.2011.04.007 . 21821478

[15] Lee SP, Souza RB, Powers CM. The influence of hip abductor muscle performance on dynamic postural stability in females with patellofemoral pain. Gait Posture. 2012;36(3):425–9. 10.1016/j.gaitpost.2012.03.024 22607792

[16] Willson JD, Kernozek TW, Arndt RL, Reznichek DA, Scott Straker J. Gluteal muscle activation during running in females with and without patellofemoral pain syndrome. Clinical biomechanics (Bristol, Avon). 2011;26(7):735–40. Epub 2011/03/11. 10.1016/j.clinbiomech.2011.02.012 . 21388728

[17] Amorim AC, Cacciari LP, Passaro AC, Silveira SRB, Amorim CF, Loss JF, et al Effect of combined actions of hip adduction/abduction on the force generation and maintenance of pelvic floor muscles in healthy women. PLoS One. 2017;12(5):e0177575 10.1371/journal.pone.0177575 . 28542276 PMC5443498

[18] Chen TB, Chou LS. Impacts of Muscle Strength and Balance Control on Sit-To-Walk and Turn Durations in the Timed up and Go Test. Arch Phys Med Rehabil. 2017 10.1016/j.apmr.2017.04.003 PubMed PMID: 28465222. 28465222

[19] Gafner S, Bastiaenen CHG, Terrier P, Punt I, Ferrari S, Gold G, et al Evaluation of hip abductor and adductor strength in the elderly: a reliability study. Eur Rev Aging Phys Act. 2017;14:5 10.1186/s11556-017-0174-6 ; PubMed Central PMCID: PMCPMC5404282. 28450961 PMC5404282

[20] Kindel C, Challis J. Joint moment-angle properties of the hip abductors and hip extensors. Physiother Theory Pract. 2017:1–8. 10.1080/09593985.2017.1323357 . 28509596

[21] Paul DJ, Nassis GP. Testing strength and power in soccer players: the application of conventional and traditional methods of assessment. Journal of strength and conditioning research. 2015;29(6):1748–58. Epub 2014/12/30. 10.1519/JSC.0000000000000807 . 25546446

[22] Drouin JM, Valovich-mcLeod TC, Shultz SJ, Gansneder BM, Perrin DH. Reliability and validity of the Biodex system 3 pro isokinetic dynamometer velocity, torque and position measurements. European Journal of Applied Physiology. 2004;91(1):22–9. 10.1007/s00421-003-0933-0 14508689

[23] Cuthbert SC, Goodheart GJ Jr. On the reliability and validity of manual muscle testing: a literature review. Chiropractic & osteopathy. 2007;15:4 Epub 2007/03/08. 10.1186/1746-1340-15-4 ; PubMed Central PMCID: PMCPMC1847521. 17341308 PMC1847521

[24] Jackson SM, Cheng MS, Smith AR Jr., Kolber MJ. Intrarater reliability of hand held dynamometry in measuring lower extremity isometric strength using a portable stabilization device. Musculoskeletal science & practice. 2017;27:137–41. Epub 2016/08/01. 10.1016/j.math.2016.07.010 . 27476066

[25] Stark T, Walker B, Phillips JK, Fejer R, Beck R. Hand-held Dynamometry Correlation With the Gold Standard Isokinetic Dynamometry: A Systematic Review. PM&R. 2011;3(5):472–9. 10.1016/j.pmrj.2010.10.025. 21570036

[26] Hansen EM, McCartney CN, Sweeney RS, Palimenio MR, Grindstaff TL. Hand‐held Dynamometer Positioning Impacts Discomfort During Quadriceps Strength Testing: A Validity and Reliability Study. International Journal of Sports Physical Therapy. 2015;10(1):62–8. PMC4325289. 25709864 PMC4325289

[27] Cahalan TD, Johnson ME, Liu S, Chao EY. Quantitative measurements of hip strength in different age groups. Clin Orthop Relat Res. 1989;(246):136–45. . 2766602

[28] Click Fenter P, Bellew JW, Pitts TA, Kay RE. Reliability of stabilised commercial dynamometers for measuring hip abduction strength: a pilot study. Br J Sports Med. 2003;37(4):331–4. Epub 2003/08/02. 10.1136/bjsm.37.4.331 ; PubMed Central PMCID: PMCPMC1724659. 12893719 PMC1724659

[29] Frese E, Brown M, Norton BJ. Clinical reliability of manual muscle testing. Middle trapezius and gluteus medius muscles. Phys Ther. 1987;67(7):1072–6. . 3602100 10.1093/ptj/67.7.1072

[30] Hayes KW, Falconer J. Reliability of hand-held dynamometry and its relationship with manual muscle testing in patients with osteoarthritis in the knee. J Orthop Sports Phys Ther. 1992;16(3):145–9. 10.2519/jospt.1992.16.3.145 . 18796764

[31] Hislop HJ, Avers D, Brown M, Daniels L. Daniels and Worthingham's muscle testing: techniques of manual examination and performance testing 9th ed St. Louis, Mo: Elsevier; 2014 xiv, 514 p. p.

[32] Lawson A, Calderon L. Interexaminer agreement for applied kinesiology manual muscle testing. Percept Mot Skills. 1997;84(2):539–46. 10.2466/pms.1997.84.2.539 . 9106846

[33] Lourencin F, Macedo E, Scarpellini. Evaluation of hip adductor and abductor muscles using an isokinetic dynamometer. Acta Fisiatr 2012;19 (1):16–20.

[34] Schmitt WH Jr., Cuthbert SC. Common errors and clinical guidelines for manual muscle testing: "the arm test" and other inaccurate procedures. Chiropractic & osteopathy. 2008;16:16 Epub 2008/12/23. 10.1186/1746-1340-16-16 ; PubMed Central PMCID: PMCPMC2628341. 19099575 PMC2628341

[35] Thorborg K, Serner A, Petersen J, Madsen TM, Magnusson P, Holmich P. Hip adduction and abduction strength profiles in elite soccer players: implications for clinical evaluation of hip adductor muscle recovery after injury. The American journal of sports medicine. 2011;39(1):121–6. Epub 2010/10/12. 10.1177/0363546510378081 . 20929931

[36] Widler KS, Glatthorn JF, Bizzini M, Impellizzeri FM, Munzinger U, Leunig M, et al Assessment of hip abductor muscle strength. A validity and reliability study. J Bone Joint Surg Am. 2009;91(11):2666–72. 10.2106/JBJS.H.01119 . 19884441

[37] Farrell M, Richards JG. Analysis of the reliability and validity of the kinetic communicator exercise device. Med Sci Sports Exerc. 1986;18(1):44–9. . 3959863

[38] Bazett-Jones DM, Cobb SC, Joshi MN, Cashin SE, Earl JE. Normalizing hip muscle strength: establishing body-size-independent measurements. Arch Phys Med Rehabil. 2011;92(1):76–82. 10.1016/j.apmr.2010.08.020 21187208

[39] Jaric S, Radosavljevic-Jaric S, Johansson H. Muscle force and muscle torque in humans require different methods when adjusting for differences in body size. European Journal of Applied Physiology. 2002;87(3):304–7. 10.1007/s00421-002-0638-9 12111294

[40] Campos Jara CA, Bautista González IJ, Chirosa Ríos LJ, Martin Tamayo I, Lopez Fuenzalida AE, Chirosa Ríos IJ. Validación y fiabilidad del dispositivo Haefni Health System 1.0 en la medición de la velocidad en el rango isocinético. Cuadernos de Psicología del Deporte. 2014;14(2):91–8.

[41] Jara CC, Ríos LJC, Mayorga DJ, Rios IC, Salazar CM, Beraldo PC. Comparison of two incremental protocols for evaluation of hip extension. Fisioterapia em Movimento. 2017;30:133–140

[42] Canadian Society for Exercise Physiology (CSEP). Physical activity readiness questionnaire PAR-Q. [Internet]. 2002 Available: http://www.csep.ca/cmfiles/publications/parq/par-q.pdf

[43] Lukaski HC, Bolonchuk WW, Hall CB, Siders WA. Validation of tetrapolar bioelectrical impedance method to assess human body composition. J Appl Physiol (1985). 1986;60(4):1327–32. Epub 1986/04/01. 3700310. 10.1152/jappl.1986.60.4.1327 3700310

[44] Bemben MG, Clasey JL, Massey BH. The effect of the rate of muscle contraction on the force-time curve parameters of male and female subjects. Res Q Exerc Sport. 1990;61: 96–99. 10.1080/02701367.1990.10607484 2091174

[45] Mukaka MM. Statistics corner: A guide to appropriate use of correlation coefficient in medical research. Malawi medical journal: the journal of Medical Association of Malawi. 2012;24(3):69–71. Epub 2013/05/03. 23638278; PubMed Central PMCID: PMCPMC3576830. PMC3576830 23638278

[46] Shrout PE, Fleiss JL. Intraclass correlations: uses in assessing rater reliability. Psychological bulletin. 1979;86(2):420–8. Epub 1979/03/01. 18839484. 10.1037//0033-2909.86.2.420 18839484

[47] Koo TK, Li MY. A Guideline of Selecting and Reporting Intraclass Correlation Coefficients for Reliability Research. Journal of Chiropractic Medicine. 15(2):155–63. 10.1016/j.jcm.2016.02.012 27330520 PMC4913118

[48] Atkinson G, Nevill AM. Statistical methods for assessing measurement error (reliability) in variables relevant to sports medicine. Sports Med. 1998;26(4):217–38. 9820922. 9820922 10.2165/00007256-199826040-00002

[49] Stokes M. Reliability and Repeatability of Methods for Measuring Muscle in Physiotherapy. Physiotherapy Practice. 1985;1(2):71–6.

[50] Eliasziw M, Young SL, Woodbury MG, Fryday-Field K. Statistical methodology for the concurrent assessment of interrater and intrarater reliability: using goniometric measurements as an example. Phys Ther. 1994;74(8):777–88. Epub 1994/08/01. 8047565. 8047565 10.1093/ptj/74.8.777

[51] Bland JM, Altman DG. Comparing methods of measurement: why plotting difference against standard method is misleading. Lancet (London, England). 1995;346(8982):1085–7. Epub 1995/10/21. 7564793. 7564793 10.1016/s0140-6736(95)91748-9

[52] Weir JP. Quantifying Test-Retest Reliability Using the Intraclass Correlation Coefficient and the SEM. J Strength Cond Res. 2005;19: 231 10.1519/15184.1 15705040

[53] Sale DG. Neural adaptation to resistance training. Med Sci Sports Exerc. 1988;20(5 Suppl):S135-45. 3057313. 10.1249/00005768-198810001-00009 3057313

[54] Davies MJ, Dalsky GP. Normalizing strength for body size differences in older adults. Med Sci Sports Exerc. 1997;29(5):713–7. Epub 1997/05/01. 9140912. 9140912 10.1097/00005768-199705000-00020

[55] Meyer C, Corten K, Wesseling M, Peers K, Simon JP, Jonkers I, et al Test-retest reliability of innovated strength tests for hip muscles. PLoS One. 2013;8(11):e81149. 10.1371/journal.pone.0081149. 24260550; PubMed Central PMCID: PMCPMC3834260. 10.1371/journal.pone.0081149 PMC3834260 24260550

[56] Thorborg K, Bandholm T, Schick M, Jensen J, Holmich P. Hip strength assessment using handheld dynamometry is subject to intertester bias when testers are of different sex and strength. Scand J Med Sci Sports. 2013;23(4):487–93. Epub 2011/11/19. 10.1111/j.1600-0838.2011.01405.x 22092308

[57] Nadler SF, DePrince ML, Hauesien N, Malanga GA, Stitik TP, Price E. Portable dynamometer anchoring station for measuring strength of the hip extensors and abductors. Arch Phys Med Rehabil. 2000;81(8):1072–6. Epub 2000/08/16. 10943757. 10943757 10.1053/apmr.2000.7165

