# Supplementary figures and images for: Validity and reliability of evaluating hip abductor strength using different normalization methods in a functional electromechanical device
Source: PLoS One. 2018 Aug 20;13(8):e0202248. doi: 10.1371/journal.pone.0202248 (PMC6101381; doi:10.1371/journal.pone.0202248)

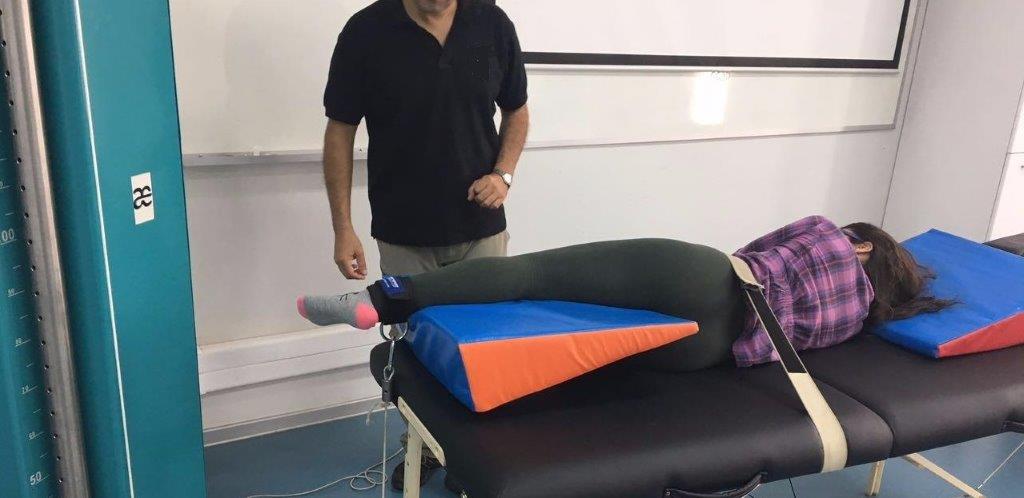

Supplement: S1 Fig — (JPG) [file pone.0202248.s002.jpg]

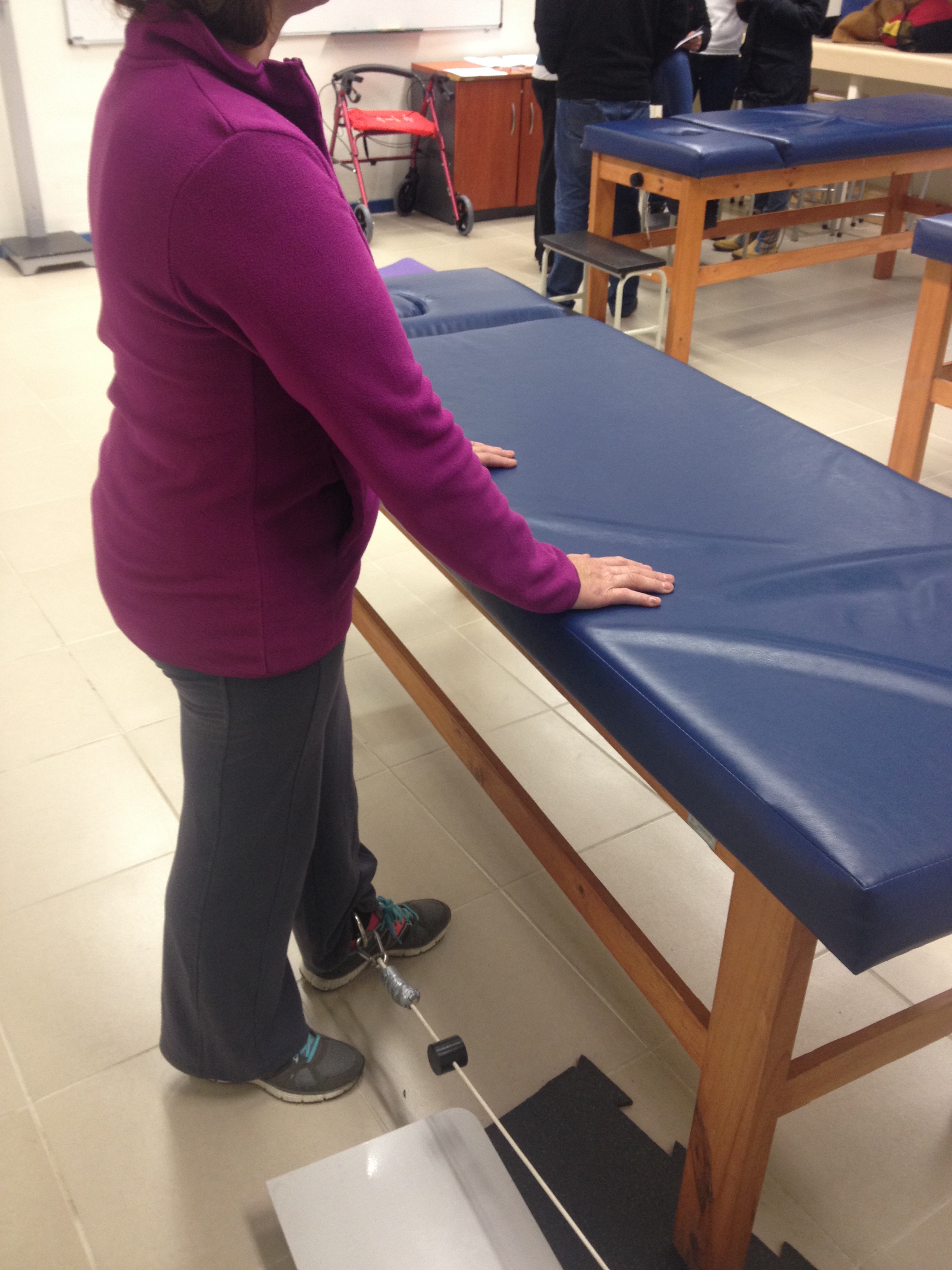

Supplement: S2 Fig — (JPG) [file pone.0202248.s003.jpg]

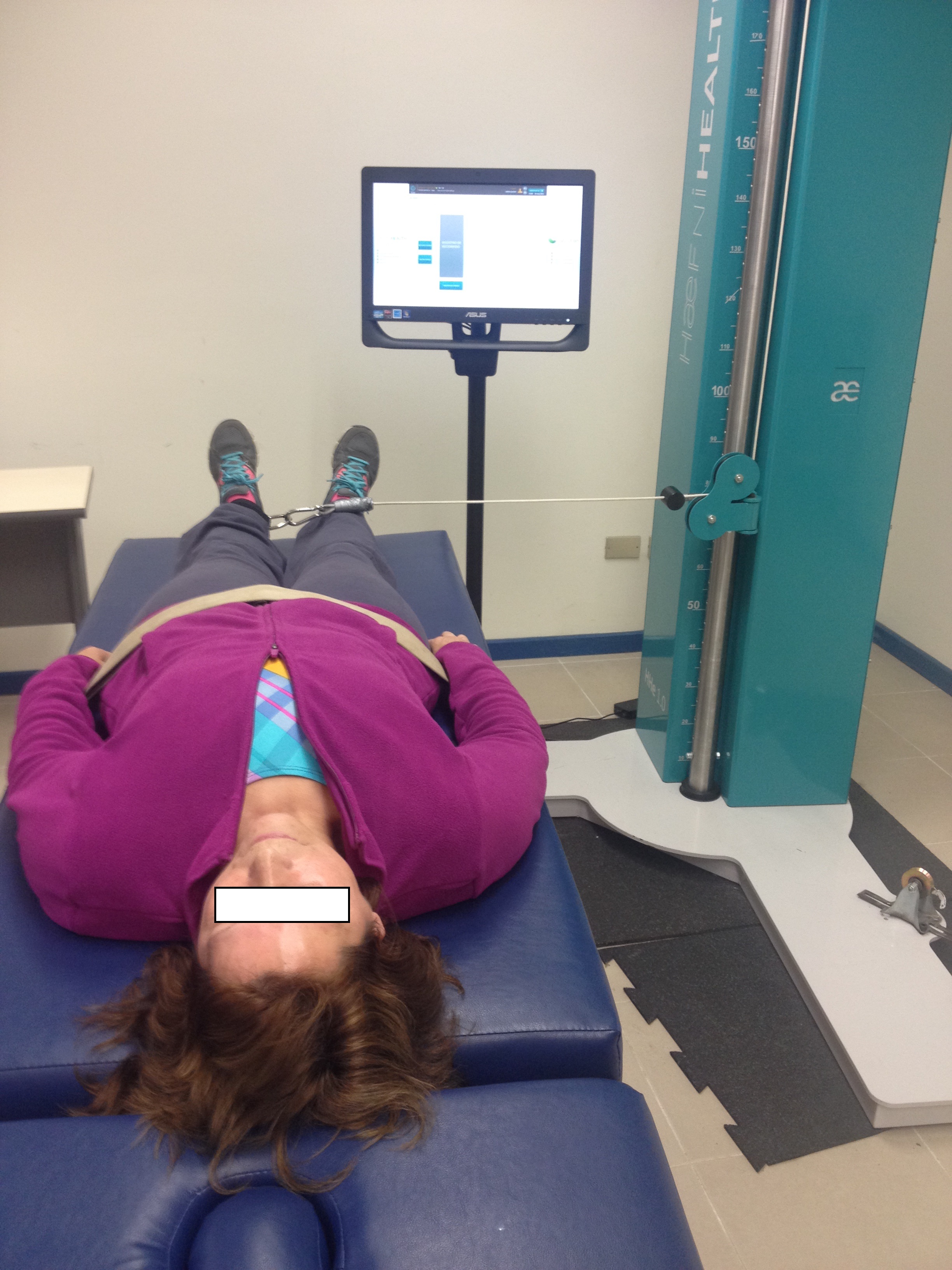

Supplement: S3 Fig — (JPG) [file pone.0202248.s004.jpg]

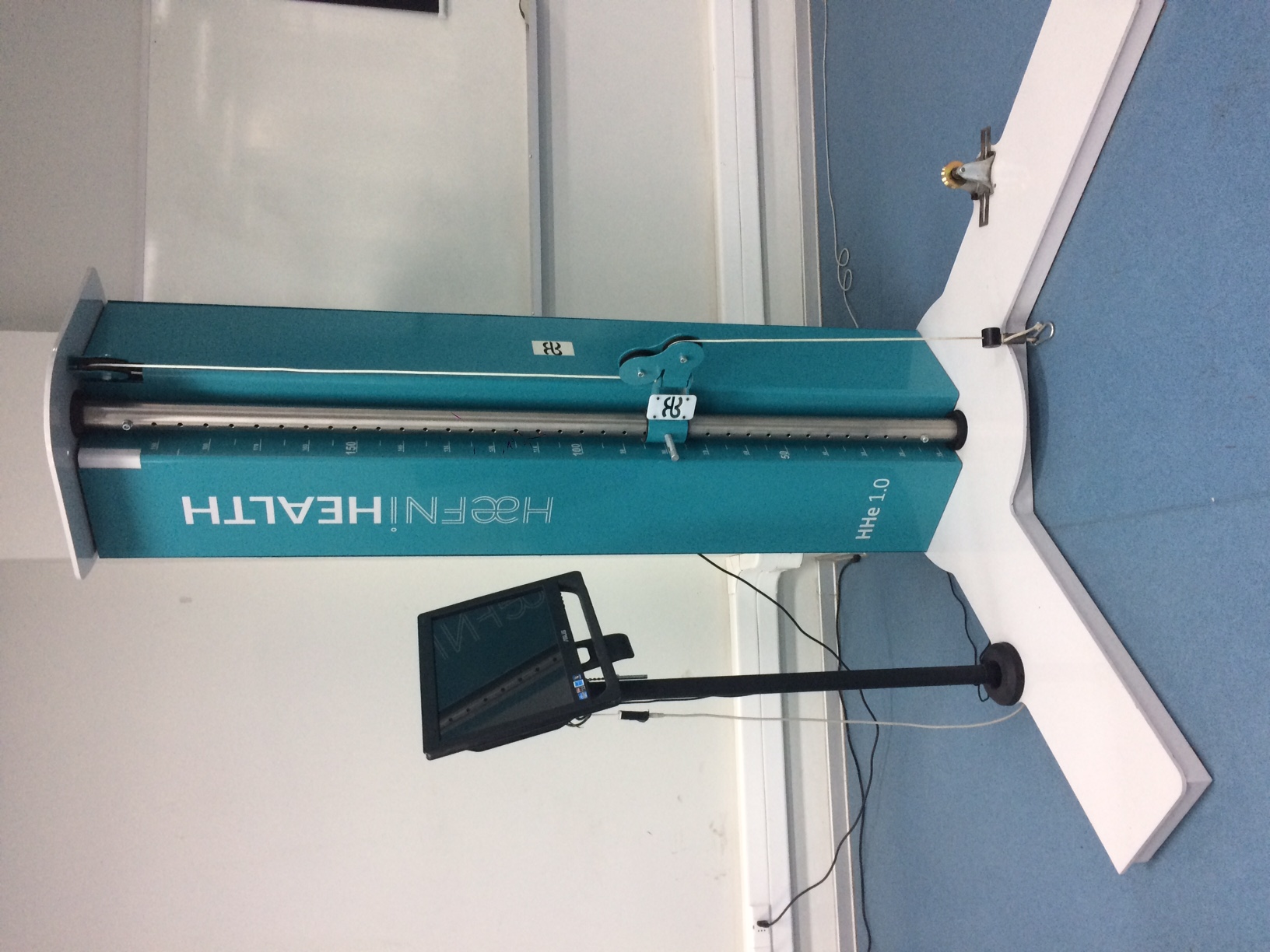

Supplement: S4 Fig — (JPG) [file pone.0202248.s005.JPG]
